# Endoscopic measurement of the size of gastrointestinal polyps using an electromagnetic tracking system and computer vision-based algorithm

**DOI:** 10.1007/s11548-023-03011-z

**Published:** 2023-08-19

**Authors:** Nazanin Safavian, Simon K. C. Toh, Martino Pani, Raymond Lee

**Affiliations:** 1 Faculty of Technology, University of Portsmouth, Portsmouth, UK; 2 Department of Upper GI Surgery, Portsmouth Hospital University NHS Trust, Queen Alexandra Hospital, Portsmouth, UK

**Keywords:** Endoscopy, Polyp, Size estimation, Electromagnetic tracking system

## Abstract

**Purpose:**

Polyp size is an important factor that may influence diagnosis and clinical management decision, but estimation by visual inspection during endoscopy is often difficult and subject to error. The purpose of this study is to develop a quantitative approach that enables an accurate and objective measurement of polyp size and to study the feasibility of the method.

**Methods:**

We attempted to estimate polyp size and location relative to the gastro-oesophageal junction by integrating data from an electromagnetic tracking sensor and endoscopic images. This method is based on estimation of the three-dimensional coordinates of the borders of the polyp by combining the endoscope camera position and the corresponding points along the polyp border in endoscopic images using a computer vision-based algorithm. We evaluated the proposed method using a simulated upper gastrointestinal endoscopy model.

**Results:**

The difference between the mean of ten measurements of one artificial polyp and its actual size (10 mm in diameter) was 0.86 mm. Similarly, the difference between the mean of ten measurements of the polyp distance from the gastroesophageal junction and its actual distance (~ 22 cm) was 1.28 mm. Our results show that the changes in camera positions in which the images were taken and the quality of the polyp segmentation have the most impact on the accuracy of polyp size estimation.

**Conclusion:**

This study demonstrated an innovative approach to endoscopic measurements using motion tracking technologies and computer vision and demonstrated its accuracy in determining the size and location of the polyp. The observed magnitude of error is clinically acceptable, and the measurements are available immediately after the images captured. To enhance accuracy, it is recommended to avoid identical images and instead utilise control wheels on the endoscope for capturing different views. Future work should further evaluate this innovative method during clinical endoscopic procedures.

## Introduction

Stomach and colorectal cancers globally were the fifth and third most common forms of cancer and the fourth and second most deadly cancer worldwide in 2020 [1]. Gastric or colorectal lesions such as polyps can potentially transform into cancer. Optimum management of detected polyps can increase the chance of diagnosing early cancer when it is still curable. Based on clinical guidelines, an accurate measurement of a polyp size found in both colonoscopy and gastroscopy is among the factors that affect clinical management. The size of a polyp is positively correlated with the risk of malignancy [2] and plays a role in selecting between resection or follow-up observation [3], determining the follow-up intervals [4] or the optimum resection technique [5]. Visual estimation made by endoscopists is subject to high inter/intra-observer variability [6] and can cause underestimation [7] or overestimation [8]. Histopathologic measurement is only available if polypectomy is part of the procedure, which might be more objective and reproducible [9]; however, some unavoidable issues such as lifting the polyp with submucosal fluid, resecting the rim of the normal tissue and shrinkage after polypectomy can make it inaccurate [10]. Erroneous endoscopic measurements affect clinical decisions adversely, whether it is underestimating the cancer risk and inappropriate surveillance interval recommendations [11] or lower rates of complete and curative resections [12]. Despite the importance of polyp size in a setting of an optimum treatment and cancer risk assessment, there is not any gold standard measuring technique in current clinical practice.

To tackle this problem, the use of a graduated endoscopic device has been widely proposed, such as a ruler snare [13], disposal graduated biopsy forceps [14] or a colonoscopy cap [15]. The limitations of being time-consuming and subjective aside, using a device might be only applicable up to a certain size [15], and the performance highly depends on whether the device can appropriately align with the polyp.

Several studies have proposed a deep learning-based approach, especially convolutional neural networks (CNN), to overcome this difficulty to classify polyps into different size groups [16, 17] or measure the direct size using a reference such as an adjacent vessel network [18]. Deep learning requires a massive amount of training data. This can be challenging in medical applications due to limited and small-scale data availability. On the other hand, the performance of the model might be affected when the model is used for data which is different from the training dataset in terms of application (e.g. colonoscopy vs. gastroscopy), quality (e.g. curated dataset) or the endoscopy manufacturer.

There is a need to provide an objective, accurate, reliable and convenient measurement technique that can be used during the endoscopic assessment. Adding a device to the endoscope can meet these criteria; however, the claimed benefits need to justify the change. For instance, the introduction of a laser emitter [19] allowed a mean error percentage of 5.3 ± 5.5 in measuring an artificial polyp; an optical probe brought an absolute measurement error of around 1 mm for three real colon polyps [20], and a pattern projector made it possible to obtain a median estimation error of 1.5 mm with IQR (interquartile range) of 1.67 mm on created polyps in ex vivo stomach [21].

The purpose of this paper is to present a newly developed quantitative method for providing the size of a polyp and its location in an endoscopy application. The method is based on an electromagnetic tracking sensor that can be embedded into a conventional endoscope combined with a computer vision-based algorithm. The efficiency of the proposed method was evaluated thereafter using an upper gastrointestinal (GI) model with an artificial polyp. To understand the fundamental performance and accuracy of the proposed method, testing on a simulated environment before testing in a real endoscopy procedure is necessary as the actual values are measurable in the former while there is no gold standard measuring technique in the latter.

## Materials and methods

The proposed quantitative method is based on a synchronous acquisition of both images and poses of the camera scope during the endoscopy procedure. The suggested methodology then requires:

1. Estimating the camera scope characteristics (camera calibration)
2. A system to detect the position and the orientation of the camera scope when an image of a polyp is acquired
3. A procedure based on computer vision operations to identify the corresponding points on the polyp border in image pairs

**Fig. 1:**
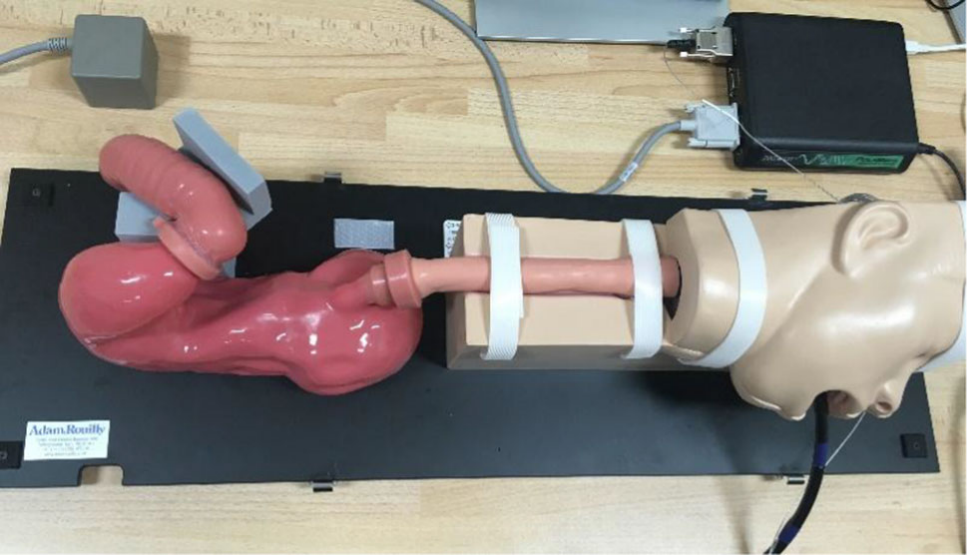
Koken EGD (EsophagoGastroDuodenoscopy) Simulator

For the evaluation of the proposed method, an artificial upper gastrointestinal model (Koken EGD Simulator, GTSimulators, Davie, Florida, USA)^1^ was used in which a rounded shape artificial polyp (10 mm in diameter) was placed in the antrum part of the stomach Fig. 1.

### Tracking system

**Fig. 2:**
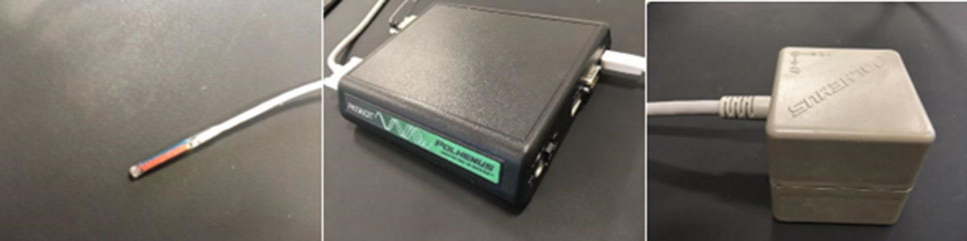
Electromagnetic tracking system. Left: Microsensor; middle: processing unit; right: TX2, the electromagnetic field generator

An electromagnetic tracking system^2^ consists of a processing unit (PATRIOT), an electromagnetic sensor (Micro sensor Ø1.8 mm) and an electromagnetic field generator (TX2) Fig. 2. The sensor has been attached to an endoscope (Pentax EPK-i) and provides 6DoF (degrees-of-freedom) for its position and orientation with respect to the TX2 coordinate system.

**Fig. 3:**
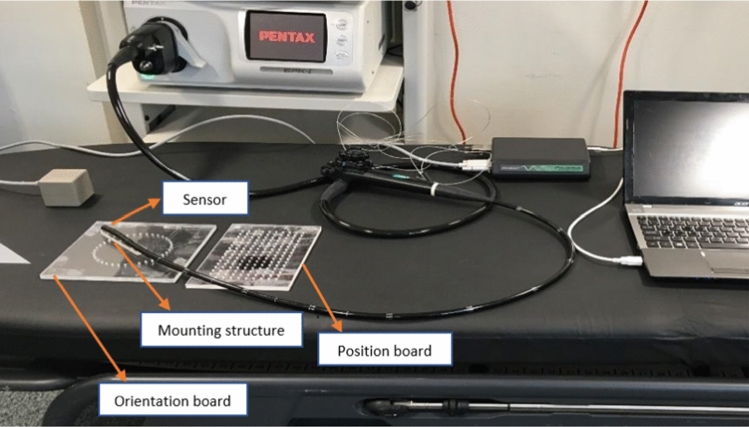
Set-up for testing the accuracy of the tracking system in the target environment

The accuracy of electromagnetic tracking systems can be affected by distorted environments in terms of ferromagnetic material interference. For this reason, the accuracy of the sensor was tested in the target environment on the same bed used in the endoscopy room (Fig. 3). The sensor was attached to the endoscope tube and was placed on a mounting structure which can move on two boards for position and orientation, similar to [22]. The position board was a known grid of 10 × 16 with a distance of 10 mm, and the orientation board was a circular shape grid with 36 steps of 10° each.

### Calibration

Two offline calibrations were performed, and the estimated parameters were then used in the computer vision-based algorithm. The first calibration, called camera calibration, aims to estimate the camera projection matrix* P*, which presents the relationship between the coordinates (*X*,* Y*,* Z*) of a point $\tilde{X}$ in a 3D scene and the coordinates (*u*, v) of its projection $\tilde{x}$ into the image plane. Here, the calibration was performed by considering the pinhole camera model according to [23] and the lens distortion model based on [24].

**Fig. 4:**
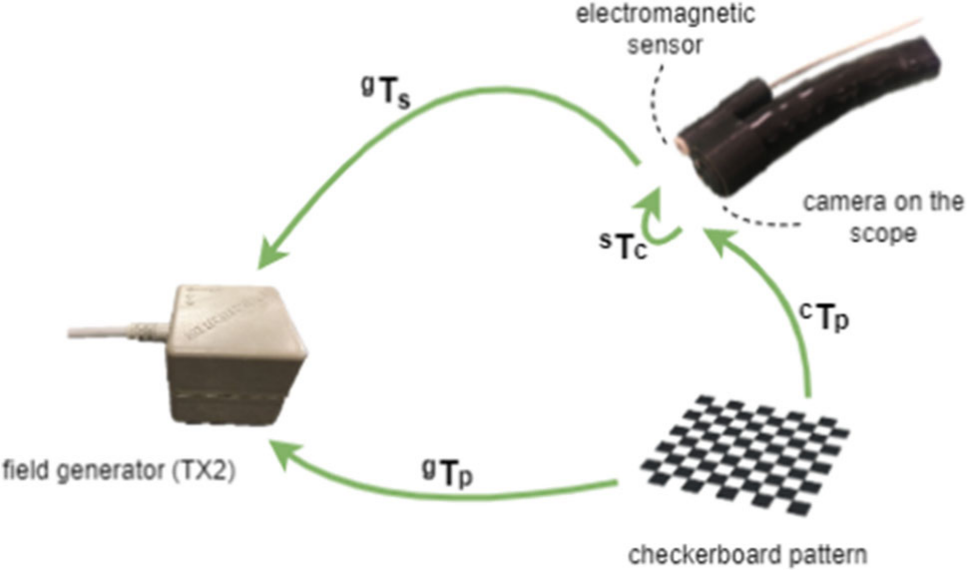
Different transformations in hand-eye calibration. ^g^*T*_s_: transformation between sensor and field generator coordinate systems, ^g^*T*_p_: transformation between checkerboard pattern and field generator coordinate systems, ^s^*T*_c_: transformation between sensor and endoscope's camera coordinate systems, ^c^*T*_p_: transformation between checkerboard pattern and endoscope's camera coordinate systems

The second calibration, called hand-eye calibration, aims to estimate the transformation (rotation and translation) matrix (^s^*T*_c_) between the camera coordinate system and the sensor coordinate system (Fig. 4). This transformation remained unchanged as the sensor's location was fixed with respect to the camera endoscope using a 3D printed cover.

The process of this calibration includes capturing multiple images of a checkerboard pattern and, at the same time, recording the corresponding sensor outputs for each pose while the checkerboard pattern is fixed with respect to the electromagnetic field generator. In Fig. 3, the transformation matrices ^g^*T*_p_ and ^s^*T*_c_ are fixed, while ^c^*T*_p_ and ^g^*T*_s_ will change in each pose. The hand-eye calibration can be mathematically formulated as a homogeneous transformation [25]:

1 $$AT=TB$$

where *A*, *B* and *T* are homogenous 4 × 4 transform matrices. *A* (*R*_a_, *t*_a_) represents the motion of the endoscope camera reference frame between two poses calculated from extrinsic parameters using the checkerboard pattern, and *B*(*R*_b_, *t*_b_) describes the motion of the sensor structure reference frame between two poses calculated from sensor recordings. *T*(*R*_t_, *t*_t_) is the required transform matrix between the camera and sensor reference coordinate systems. For each motion *i*, Eq. (1) can be split into two equations as follows:

2 $${R}_{ai}{R}_{t}={R}_{t}{R}_{bi}$$

3 $${R}_{ai}{t}_{t}+{t}_{ai}={R}_{t}{t}_{bi}+ {t}_{t}$$

The Open-source Computer Vision library (OpenCV) was used for solving Eq. (1), in which the solution explained in [26] led to more robust results on our dataset. Based on [26], Eq. (2) and (3) can be rewritten as the homogeneous linear system for all motion i as follows:

4 $$ \left[\begin{array}{ccc} I_{9} - R_{ai} \otimes R_{bi}& 0_{9 \times 3}\\ I_{3} \otimes \left( {t_{bi} } \right)^{T}& I_{3} -R_{ai}  \end{array}\right]\left[\begin{array}{ccc} vec\left( {R_{t}} \right)\\ t_{t} \end{array} \right] = \left[\begin{array}{ccc}0_{9 \times 1}\\ t_{ai}\end{array}\right] $$

where ⨂ is the Kronecker product [27]. This homogeneous linear system will then be solved by the linear least-square minimisation technique. In this study, 50 poses from a checkerboard pattern (8 × 9) with a square size of 3 mm were used for both calibrations. Apart from which method is used to solve Eq. (1), the poses as the inputs for the hand-eye calibration process also play an essential role in acquiring accurate estimation. We considered the suggestions proposed in [28] as follows:

1. Maximise the angle between rotation axes of relative movements
2. Maximise the rotation angle of relative movements
3. Minimise the distance between the optical centre of the camera and the calibration pattern
4. Minimise the distance between the sensor coordinate system positions

While suggestions number 3 and 4 are easy to apply while capturing the poses, suggestions number 1 and 2 can be met by selecting a subset of the poses after data were acquired [29].

### Computer vision-based algorithm

**Fig. 5:**
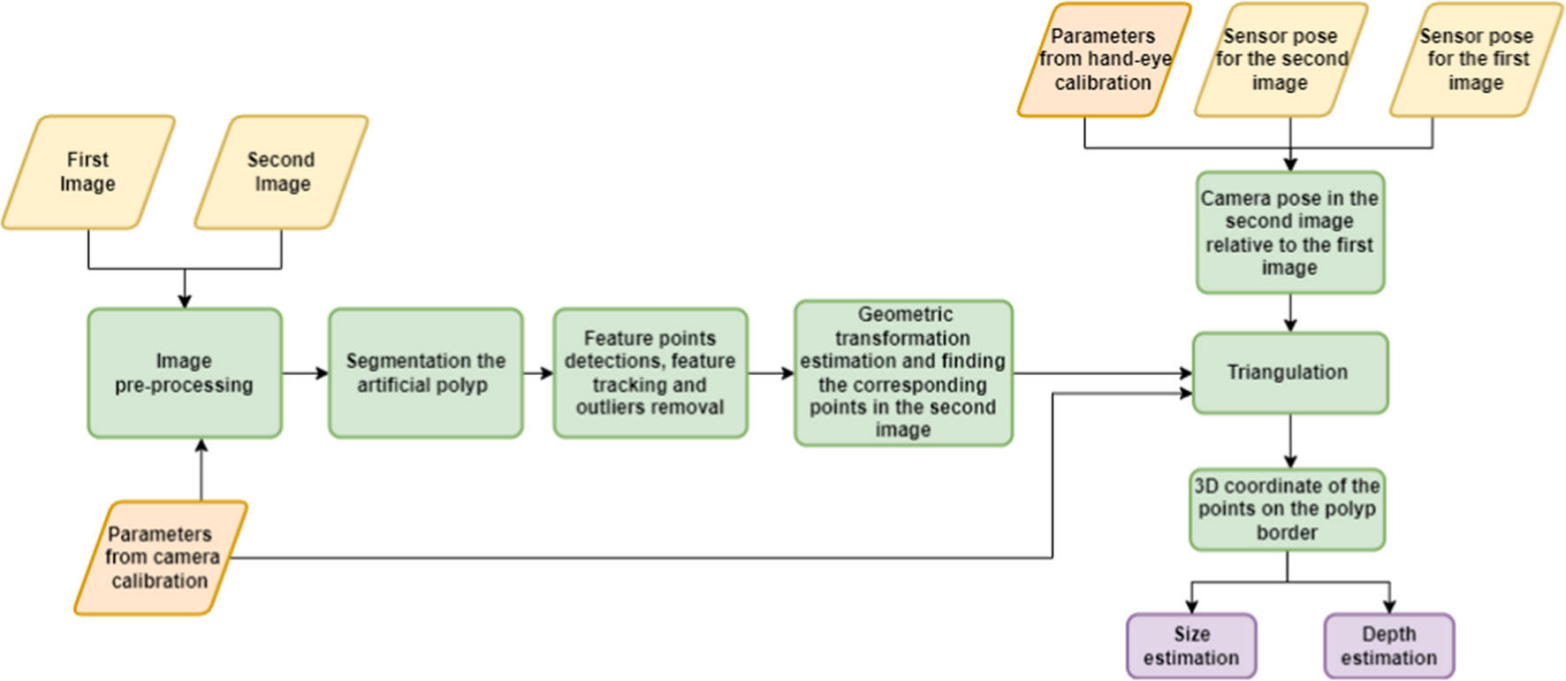
The flow chart of the proposed computer vision-based algorithm

The flow chart of the proposed computer vision-based algorithm implemented in MATLAB is shown in Fig. 5. The inputs for the algorithm are: (1) the estimated parameters from the calibrations and (2) two non-identical endoscopic images of a target object (an artificial polyp) as well as (3) the corresponding sensor position outputs. An existing pushbutton on the control body of the endoscope was used to simultaneously trigger the image capturing and recording of the sensor outputs. First, some image pre-processing was applied to these two images, such as contrast adjustment followed by correction of lens distortion using estimated intrinsic parameters from camera calibration.

Second, in the first image, the border of the artificial polyp was identified as a region of interest (ROI) using an automatic segmentation method. The method was based on a canny edge detection technique applied on the V-channel of HSV colour space and utilising the morphologic characteristic of the artificial polyp, such as eccentricity. Third, considering a margin around this ROI, image feature points were detected using state-of-the-art feature detection techniques which are invariant to scale and rotation changes, such as SURF, SIFT, BRISK and ORB [30] and subsequently, their feature descriptions were extracted. Next, these feature points were tracked in the second image based on [31]. Outliers of the matched features were eliminated considering the epipolar constraint [32]. Then, using these matched pair points, the geometric linear affine transformation between these two images was estimated and applied to all points on the ROI in the first image to identify the corresponding points in the second image.

Additionally, using the estimated transformation from the hand-eye calibration and the sensor recordings in two poses, the relative camera pose was computed with respect to the camera coordinate system. Lastly, by having the matched points of all the points on the polyp border in two images, intrinsic camera matrix and relative camera pose, 3D coordinates of all the points with respect to the camera coordinate system were obtained using triangulation [32]. An ellipse was fitted to these 3D coordinates, and the longest length of this ellipse was considered as the polyp size. The depth between the artificial polyp and the camera scope was considered the *z*-coordinate of the centre of the fitted ellipse.

### Evaluation of the quantitative method

#### Sensitivity of the polyp size measurements

We evaluated the sensitivity of the measurements by studying the impact of several factors on the accuracy (relative error) of assessing the polyp size using the proposed method, including the type of endoscope movements, the distance from the electromagnetic source and the endoscope, the distance from the camera scope and the polyp (depth) and the segmentation quality of identifying the polyp border. The following factors were studied.

##### The impact of endoscope movements

Six different types of movements based on the relative positions of the camera endoscope were defined while capturing the image pairs Table 1. The rotation here refers to the angle of rotation computed from the rotation matrix.

**Table 1:** Six different types of relative movements of the endoscope

Movements type	Description
0	Movements with displacement less than 3 mm
1	Movements without changing the depth (less than 2 mm) with rotation less than 20 degrees
2	Movements without changing the depth (less than 2 mm) with rotation larger than 20 degrees
3	Movements with changing the depth with small rotation (less than 5 degrees)
4	Movements with changing the depth with rotation between 5 and 20 degrees
5	Movements with changing the depth with rotation larger than 20 degrees

A total of 30 image pairs were captured from the artificial polyp, including five image pairs presenting one of each six types of movement Fig. 7.

##### The impact of distance from the electromagnetic source

In this test, the movement of type 1 was repeated sixteen times, and each time the electromagnetic source was replaced to a further position (~ 18–52 cm) Fig. 8.

##### The impact of depth

The movement type 1 repeated 17 times while the distance from the electromagnetic source was less than 30 cm. Each time the image pairs from the artificial polyp were captured at different depths (distance from the endoscope with respect to the polyp) in a range of 7 to 58 mm Fig. 9.

##### The segmentation quality impact

One image pair from movement type 1 was selected while the distance from the electromagnetic source was less than 30 cm, and the depth was around 26.7 mm. For the perfect polyp segmentation, the percentage error of estimated polyp size estimation was 0.4%. The polyp was segmented 20 times in the first image manually with different levels of overestimation (10 times) and underestimation (10 times) segmentations. To evaluate the quality of the segmentation, the Dice similarity coefficient (DSC) was used Fig. 10.

#### Accuracy of the polyp size and location measurement

The endoscope was inserted ten times inside the same upper GI model. For each measurement, as soon as the endoscope had passed the *Z*-line anatomical landmark before the gastroesophageal junction, the sensor output was recorded. Then inside the stomach, four images with their corresponding sensor outputs were recorded from different angles of the artificial polyp. The longest length of the polyp and the distance from the polyp centre to the Z line were estimated using the quantitative method and compared with the actual values.

## Result

### Tracking system

**Fig. 6:**
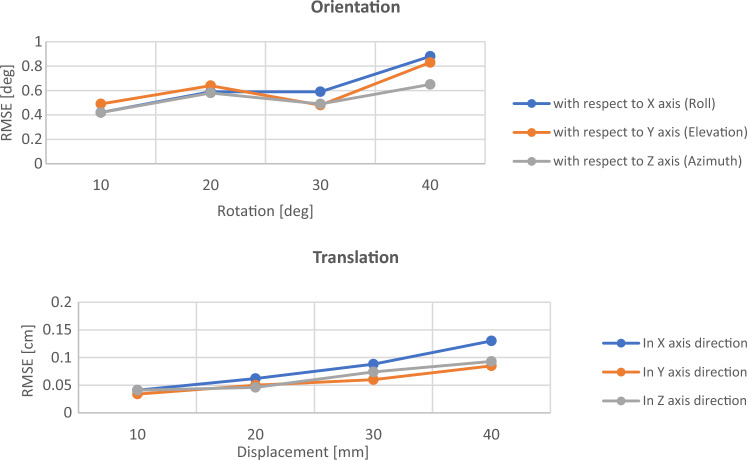
Multiple measurements were recorded while the distances of the midpoint of the displacement from the electromagnetic source were in the range of 30 cm to 46 cm. The test was repeated for 10, 20, 30 and 40 mm displacements as well as 10°, 20°, 30° and 40° rotations

The accuracy of the tracking system was tested using the setting explained in the method section. The root means square error (RMSE) for different translations of 10, 20, 30 and 40 mm and different rotations of 10, 20, 30 and 40 degrees with respect to axes of the electromagnetic source coordinate system were shown in Fig. 6. Considering all axes and different levels of sensor movements, the overall RMSE for translation and rotation were 0.73 mm and 0.61 degrees, respectively.

### Sensitivity of the polyp size measurements

**Fig. 7:**
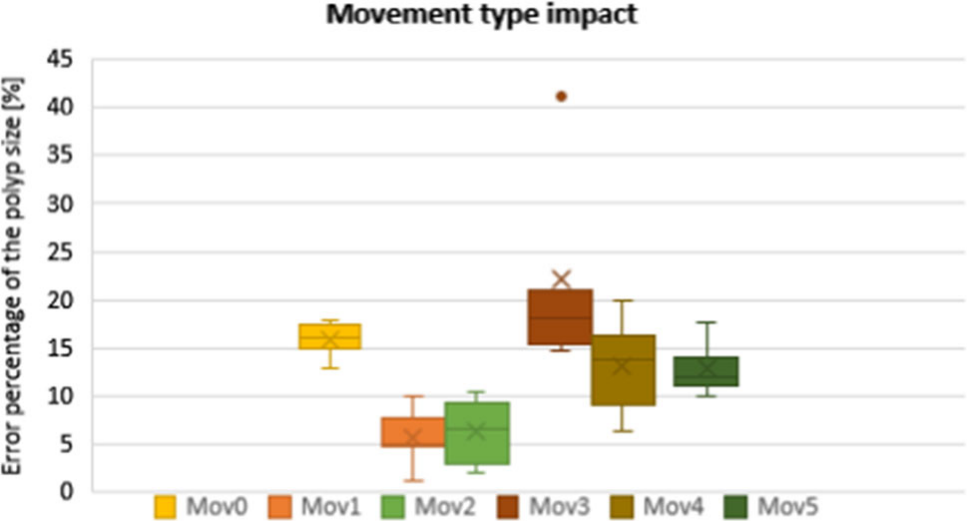
Error percentage of the polyp size estimation for different endoscope movements. The line and the cross in the box show the median and the mean of the error percentage, respectively. The dots represent outliers

Figure 7 shows the Box-and-Whisker plot that was used to compare the relative error in assessing the polyp size related to each movement type. According to Fig. 7, movement types 1 and 2 have a relatively lower median and mean error percentage of less than 7%. The median and mean of error percentage increases for movements number 4 and 5 (between 10 and 15%) and is relatively higher for movement number 0 and 3 (more than 15%).

**Fig. 8:**
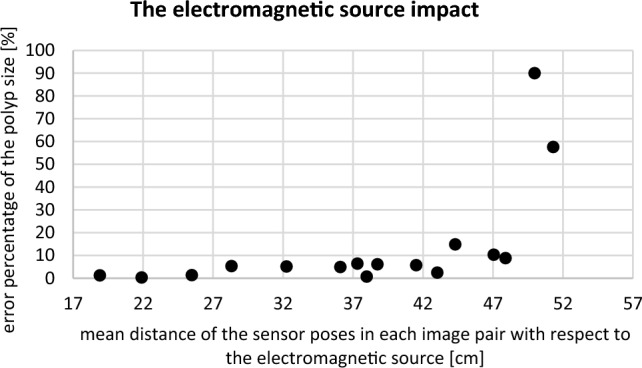
The error percentage of the polyp size estimation against the different distances of the sensor poses with respect to the electromagnetic source in centimetres

Figure 8 shows the percentage of error in estimated polyp size against the mean distance of the sensor poses with respect to the electromagnetic source in centimetres. The relative error remained below 5% for distances between the sensor and the electromagnetic source up to 36 cm. It can be seen that as the distance increased to approximately 48 cm, the relative error increased slightly. For a distance greater than 48 cm, the percentage of the error incremented dramatically.

**Fig. 9:**
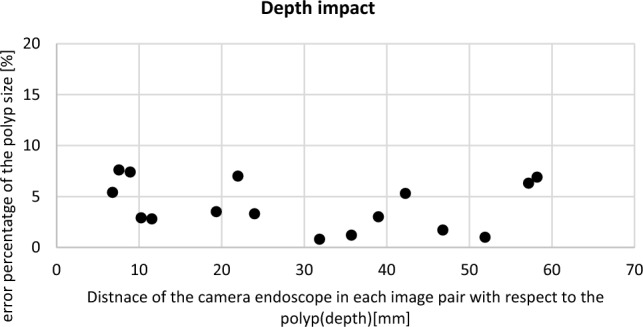
The error percentage of the polyp size estimation against different depths in millimetres

Figure 9 shows the percentage of the error in polyp size estimation against the depth in millimetres. According to Fig. 9, the error is less than 6% when the distance between the endoscope and the polyp is approximately in the range of 25–55 mm. A depth of less than 10 mm increases the error due to the increment of camera distortion level. The increased error for a depth of more than 55 mm can be related to the smaller polyp region where the feature detection and extraction were computed.

**Fig. 10:**
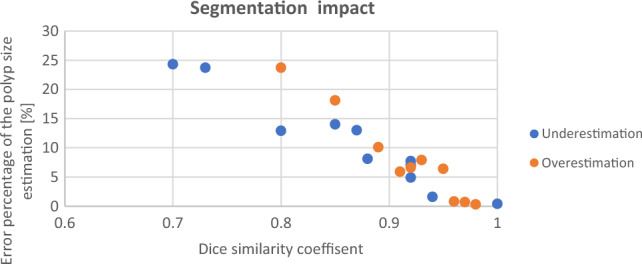
Error percentage of the polyp size estimation against different segmentation qualities in DSC

Figure 10 shows the error percentage of polyp size estimation against the segmentation quality presented in DSC for both under- and over-estimations. According to Fig. 10, the quality of the segmentation to achieve the error percentage in polyp size estimation less than 10% should have a DSC of 0.9 or higher. No difference was observed comparing the overestimation and underestimation segmentation.

### Accuracy of the polyp size and location measurement

**Fig. 11:**
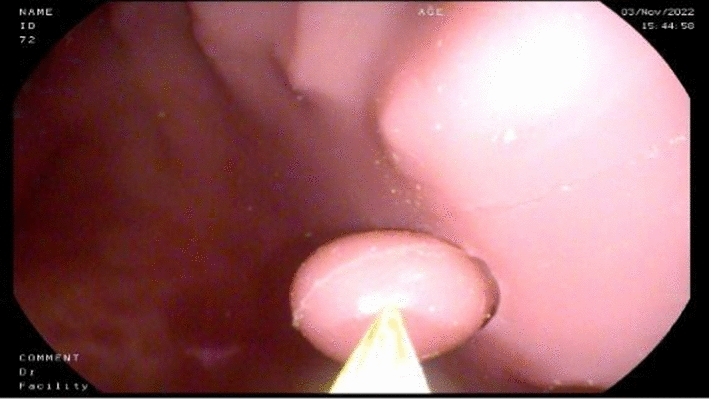
The artificial polyp and the pointer attached to the surface

The actual longest length of the polyp was measured using a digital calliper (10 mm in diameter). For computing the actual distance between the Z line and the polyp, a pointer was attached to the sensor (Fig. 11) to obtain the position of its end using sensor recordings. Knowing the position of the points on the polyp surface and the Z line periphery, the actual distance between the centre of the polyp and the centre of the Z line was computed (22.357 cm).

The simulated endoscopy procedure was performed as has been explained in the methodology section. The conditions for recording these data in terms of different factors discussed in sensitivity evaluation are summarised in Table 2.

**Table 2:** Conditions for all possible image pairs in the accuracy test

Quality of automated segmentation (DSC)	> 0.9
Depth [mm]	[22.5 57.5]
Type of movements	1(95%) and 4(5%)
Distance from the electromagnetic source [cm]	31.22 ± 0.3

The quantitative method used the first two images and their corresponding sensor recordings as an input (approach A), and another time all four images (six possible image pairs) and their corresponding sensor recordings (approach B). In approach B, the estimated minimum and maximum values were excluded, and the average of the remaining four estimations was considered the final estimation. Table 3 shows the results of comparing the estimated values by quantitative method and actual values. According to Table 3, the coefficient of variation, standard deviation and standard deviation of the error percentage for both polyp size and its distance is slightly smaller in approach B than in approach A.

**Table 3:** Performance of the quantitative method in estimating the polyp size and its distance with respect to the Z line

Metrics	Approach A		Approach B	
	Polyp size in diameter	Polyp distance with respect to the Z line	Polyp size in diameter	Polyp distance with respect to the Z line
Mean [mm]	10.86	224.85	10.93	225.03
SD [mm]	0.38	2.18	0.16	1.63
The absolute difference between the mean and actual size[mm]	0.86	1.28	0.93	1.46
RMSE [mm]	0.947	2.53	0.946	2.19
Coefficient of variation [%]	3.5	0.97	1.46	0.72
Mean percentage of the error [%]	8.65 ± 3.8	0.81 ± 0.79	9.32 ± 1.64	0.87 ± 0.59

Approach A and approach B demonstrated the performance when two and four images were used for estimation, respectively

## Discussion

Despite the importance of measuring the polyp size for clinical assessment during the endoscopy procedure, establishing an accurate and objective measurement method still remains challenging. If the method is time-consuming compared to visual estimation, such as tools-based methods, it would not be widely used in clinical practice even though they can be managed to properly align the tool in order to reach the maximum potential accuracy. On the other hand, deep learning approaches do not need any devices or tools; however, they need a large number of annotated datasets and only can classify the polyp into different size groups unless a reference can be found near the polyp [18]. It is worth noting that the accuracy reported in deep learning-based studies [16, 17] shows how closely the network can perform to the method that has been utilised for training the model, whether it is a visual estimation by one expert or a consensus of multiple experts with or without using a tool. Therefore, training the model based on inaccurate visual techniques might not add value at this stage.

As shown in Fig. 6, the accuracy of the electromagnetic sensor outputs was not affected while it was attached to the endoscope, which confirms the possibility of using this tracking system in endoscopy procedures. In Fig. 8, the error percentage of polyp estimation remained stable for distances lower than 48 cm from the electromagnetic source. Therefore, placing the electromagnetic source at a distance less than 48 cm from the abdomen is recommended, and it seems achievable in endoscopy applications. As shown in Fig. 7, it is recommended that movements such as nearly pure scale (type 3) or nearly no movement (type 0) be avoided while capturing the image pairs from the polyp. This is because of the larger error in finding the intersection point of projection lines in the triangulation step related to movements such as type 0 & 3. Instead, using the two wheels on the control body of the endoscope for turning the tip of the scope laterally is recommended while trying not to change the depth significantly (type 1&2). Based on Table 3, the level of variation in measurements using approach B is smaller, which suggests that taking four images instead of two might lead to more robust measurements. As shown in Fig. 9, keeping the distance between the polyp and the endoscope less than 10 mm or more than 55 mm leads to a relatively larger error. This threshold can be different for different sizes of polyps. A general recommendation is to keep the balance between the polyp and background regions while capturing images; this means trying not to take images from a large polyp at a close distance or from a small polyp at a far distance.

The proposed method achieved the mean absolute error of less than 1 mm for a polyp having a diameter of 10 mm, which is similar to other adding device methods [19–21]. When compared to other methods, the proposed method is not affected by polyp size or tilt angle [20] or the necessity of placing the endoscope parallel to the polyp plane [19]. On the other hand, the method has minimal impact on the endoscopy procedure: as it only requires taking pictures of the polyp, which should be done anyway as part of the recommendations based on clinical guidelines. In addition, unlike other device-based studies [19–21], the proposed method can also localise the polyp with an error of nearly 1% without any relevant change to either the equipment or the procedure.

This study had some limitations. First, a simulated model and an artificial polyp that were used to evaluate the method are different from the real condition of an endoscopy procedure. The polyp used was hemispherical, which means the evaluation might be different for different morphologies of a real polyp. Second, the real polyp might not have similar texture and rigidity. Intestinal peristalsis or insufflation in vivo can make the polyp move between capturing images, and the model did not take into account such potential error. Third, the nature of the images in real endoscopy can be different and can be affected by bubbles**,** light reflection on moist tissues or any obstructions on the camera scope. Therefore, the accuracy of the image processing techniques reported here might be different for real endoscopy images. Despite the above limitations, this study provided the proof of concept of an accurate and objective method of determining polyp size and location during endoscopic examination of the upper gastrointestinal tract. The results provide confidence that the method has acceptable accuracy and feasibility. It is now in a position that should be considered for further clinical investigations in human subjects.

## Conclusion

In this paper, we developed a quantitative method to measure the size and location of a polyp during simulated endoscopy. Information from endoscopic images was combined with knowledge of the camera endoscope position extracted from the electromagnetic tracking sensor. Results show that this method can estimate polyp size and its location objectively and accurately. Future work is suggested further to evaluate this innovative method during clinical endoscopic procedures.

## Footnotes

1 Anatomical Education Models | Koken Co., Ltd. (https://www.kokenmpc.co.jp/english/products/educational_medical_models/anatomical/).

2 https://polhemus.com/applications/electromagnetics/.

## References

[1] Sung H, Ferlay J, Siegel RL, Laversanne M, Soerjomataram I, Jemal A (2021). Global cancer statistics 2020: GLOBOCAN estimates of incidence and mortality worldwide for 36 cancers in 185 countries. CA Cancer J Clin.

[2] Goddard AF, Badreldin R, Pritchard DM, Walker MM, Warren B (2010). The management of gastric polyps. Gut.

[3] Banks M, Graham D, Jansen M, Gotoda T, Coda S, Di Pietro M (2019). British society of gastroenterology guidelines on the diagnosis and management of patients at risk of gastric adenocarcinoma. Gut.

[4] Hassan C, Antonelli G, Dumonceau JM, Regula J, Bretthauer M, Chaussade S (2020). Post-polypectomy colonoscopy surveillance: European society of gastrointestinal endoscopy (ESGE) guideline–update 2020. Endoscopy.

[5] Pimentel-Nunes P, Dinis-Ribeiro M, Ponchon T, Repici A, Vieth M, De Ceglie A (2015). Endoscopic submucosal dissection: European society of gastrointestinal endoscopy (ESGE) guideline. Endoscopy.

[6] Elwir S, Shaukat A, Shaw M, Hughes J, Colton J (2017). Variability in, and factors associated with, sizing of polyps by endoscopists at a large community practice. Endosc Int Open.

[7] Moug SJ, Vernall N, Saldanha J, McGregor JR, Balsitis M, Diament RH (2010). Endoscopists’ estimation of size should not determine surveillance of colonic polyps. Colorectal Dis.

[8] Anderson BW, Smyrk TC, Anderson KS, Mahoney DW, Devens ME, Sweetser SR (2016). Endoscopic overestimation of colorectal polyp size. Gastrointest Endosc.

[9] Schoen RE, Gerber LD, Margulies C (1997). The pathologic measurement of polyp size is preferable to the endoscopic estimate. Gastrointest Endosc.

[10] Morales TG, Sampliner RE, Garewal HS, Fennerty MB, Aickin M (1996). The difference in colon polyp size before and after removal. Gastrointest Endosc.

[11] Eichenseer PJ, Dhanekula R, Jakate S, Mobarhan S, Melson JE (2013). Endoscopic mis-sizing of polyps changes colorectal cancer surveillance recommendations. Dis Colon Rectum.

[12] Shim CN, Song MK, Kang DR, Chung HS, Park JC, Lee H (2014). Size discrepancy between endoscopic size and pathologic size is not negligible in endoscopic resection for early gastric cancer. Surg Endosc.

[13] Kaz AM, Anwar A, Robinson DO, Dominitz JA. Use of a novel polyp ‘ruler snare’ improves estimation of colon polyp size. Available from: 10.1016/j.gie.2015.08.082 10.1016/j.gie.2015.08.082 26382052

[14] Jin HY, Leng Q (2015). Use of disposable graduated biopsy forceps improves accuracy of polyp size measurements during endoscopy. World J Gastroenterol WJG.

[15] Han SK, Kim H, Kim JW, Kim HS, Kim SY, Park HJ (2021). Usefulness of a colonoscopy cap with an external grid for the measurement of small-sized colorectal polyps: a prospective randomized trial. J Clin Med.

[16] Itoh H, Oda M, Jiang K, Mori Y, Misawa M, Kudo SE (2021). Binary polyp-size classification based on deep-learned spatial information. Int J Comput Assist Radiol Surg.

[17] Requa J, Dao T, Ninh A, Karnes W (2018). Can a convolutional neural network solve the polyp size dilemma? Category Award (Colorectal Cancer Prevention) Presidential Poster Award: 282. Off J Am College Gastroenterol ACG.

[18] Kwak MS, Cha JM, Jeon JW, Yoon JY, Park JW (2022). Artificial intelligence-based measurement outperforms current methods for colorectal polyp size measurement. Dig Endosc.

[19] Yoshioka M, Sakaguchi Y, Utsunomiya D, Sonoda S, Tatsuta T, Ozawa S (2021). Virtual scale function of gastrointestinal endoscopy for accurate polyp size estimation in real-time: a preliminary study. J Biomed Opt.

[20] Oka K, Seki T, Akatsu T, Wakabayashi T, Inui K, Yoshino J (2014). Clinical study using novel endoscopic system for measuring size of gastrointestinal lesion. World J Gastroenterol WJG.

[21] Visentini-Scarzanella M, Kawasaki H, Furukawa R, Bonino MA, Arolfo S, Secco GL (2018). A structured light laser probe for gastrointestinal polyp size measurement: a preliminary comparative study. Endosc Int Open..

[22] Hummel J, Figl M, Birkfellner W, Bax MR, Shahidi R, Maurer CR (2006). Evaluation of a new electromagnetic tracking system using a standardized assessment protocol. Phys Med Biol.

[23] Zhang Z (2000). A flexible new technique for camera calibration. IEEE Trans Pattern Anal Mach Intell.

[24] Heikkila J, Silven O (1997) A four-step camera calibration procedure with implicit image correction. In: Proceedings of ieee computer society conference on computer vision and pattern recognition. IEEE. pp 1106–1112

[25] Shiu YC, Ahmad S (1987) Calibration of wrist-mounted robotic sensors by solving homogeneous transform equations of the form AX= XB

[26] Andreff N, Horaud R, Espiau B (1999) On-line hand-eye calibration. In: Second international conference on 3-D digital imaging and modeling (Cat No. PR00062). IEEE pp 430–436.

[27] Brewer J (1978). Kronecker products and matrix calculus in system theory. IEEE Trans Circuits Syst.

[28] Tsai RY, Lenz RK (1989). A new technique for fully autonomous and efficient 3 d robotics hand/eye calibration. IEEE Trans Robot Autom.

[29] Schmidt J, Vogt F, Niemann H (2003) Robust hand–eye calibration of an endoscopic surgery robot using dual quaternions. In: Joint pattern recognition symposium. Springer, pp 548–56

[30] Tareen SAK, Saleem Z (2018) A comparative analysis of sift, surf, kaze, akaze, orb, and brisk. In: 2018 International conference on computing, mathematics and engineering technologies (iCoMET). IEEE. pp 1–10

[31] Tomasi C, Kanade T (1991). Detection and tracking of point. Int J Comput Vis.

[32] Hartley R, Zisserman A (2003) Multiple view geometry in computer vision. Cambridge university press.

